# Exploring cross-cultural effectiveness of internet-based depression treatment (IBAT-D) with peer-to-peer support vs. without across WEIRD and non-WEIRD samples: a research protocol for a randomized controlled trial

**DOI:** 10.1186/s13063-025-09377-6

**Published:** 2025-12-11

**Authors:** Tanya Tandon, Thomas Berger, Björn Meyer, Omar Abou Khaled, Rashmi Gupta, Chantal Martin-Soelch

**Affiliations:** 1 Unit of Clinical and Health Psychology, Department of Psychology, University of Fribourg, Fribourg, CH-1700 Switzerland; 2 Institute of Psychology, University of Bern, Bern, Switzerland; 3 Department of Research , Gaia AG, Hamburg, Germany; 4 Haute École d’Ingénierie Et d’Architecture de Fribourg, Fribourg, Switzerland; 5 Department of Humanities and Social Sciences, Cognitive and Behavioural Neuroscience Laboratory, Indian Institute of Technology Bombay, Mumbai, MH India; 6 Koita Centre for Digital Health, Indian Institute of Technology Bombay, Mumbai, MH India

**Keywords:** Depression, Randomized controlled trial, Deprexis, Reward, Social support, Peer support, Online forum, Cross-cultural, WEIRD countries, Non-WEIRD countries, India, Switzerland

## Abstract

**Background:**

Internet-based self-help interventions (IBIs) have proven effective in reducing depression, especially in high and middle-income countries, and have proven to be flexible and location-independent. However, unguided IBIs often face high dropout rates, low uptake, lower adherence, and reduced effectiveness.

**Objective:**

This study evaluates the effectiveness of a self-help program against depressive symptoms (Deprexis) with or without peer-to-peer support, focusing on cultural differences between WEIRD (Western, Educated, Industrialized, Rich, and Democratic) and non-WEIRD countries, specifically Switzerland and India. Additionally, this research explores Deprexis’s efficacy in French-speaking Switzerland, aiming to extend its benefits to this demographic. The study also investigates mood responses to monetary and social rewards using the Fribourg Reward Task to understand the relationship between depression and reward system dysfunction in a cross-cultural context.

**Methods:**

The study is a randomized controlled trial; participants with mild to moderate depression will be randomized into three groups of 80 each: Deprexis only, Deprexis with peer-to-peer support, and a control group on a waiting list. The intervention lasts 8 weeks, with measurements at baseline (T0), mid-intervention (4 weeks, T1), post-intervention (8 weeks, T2), and follow-up (3 months post-intervention, T3). Participants will complete online questionnaires on RedCap and the Fribourg Reward Task. Primary outcome: depressive symptoms at 8 weeks post-intervention. Secondary outcomes: mood responses to reward, pleasure, and social support, with moderators and mediators like anxiety, stress, quality of life, PTSD, childhood trauma, self-efficacy, and self-esteem.

**Results:**

The study, registered at ClinicalTrials.gov (NCT06480474) and Swiss National Clinical Trials (SNCTP000005917), was approved by the Ethics Committee of Vaud (CER-VD) in May 2024 (protocol date: 22.05.2024; version: 4; 2023-D0112). Recruitment began in June 2024 and is expected to end in May 2026.

**Conclusion:**

This study examines the potential of IBIs, including peer-to-peer support forums and the Deprexis program, in addressing depression globally. It investigates the extent to which cultural adaptations may be required in order to integrate globally available online mental health care into existing health systems to bridge treatment gaps and improve outcomes. Future research should explore these interventions’ effectiveness in diverse cultural settings and their long-term impacts.

**Supplementary Information:**

The online version contains supplementary material available at 10.1186/s13063-025-09377-6.

## Introduction

Before 2020, mental health issues, particularly depression, were already a significant global health burden. The COVID-19 pandemic exacerbated this crisis, with global depression rates rising to 25% in the first year [1]. Depression is often associated with widespread stigma, discrimination, and human rights violations; for instance, lack of proper education, lack of access to information, and inequalities around access to quality mental health services [2–4]. Even before the pandemic, access to effective mental health care was limited, with 71% of individuals with depression worldwide not receiving treatment, showing stark disparities between WEIRD (Western, Educated, Industrialized, Rich, and Democratic) countries (70% treated) and non-WEIRD countries (12% treated) [5]. Despite the availability of effective treatments like psychotherapy, a global treatment gap of 44% persists [3]. Barriers include difficulty accessing services, shortages of mental health professionals, stigma, and cultural beliefs [6].

In Switzerland, the prevalence of depression is 15% [7] with a 51% treatment gap [8]. Addressing these challenges requires prioritizing accessible mental health care across all income levels. Internet-based self-help interventions (IBIs) have proven effective in symptom reduction, offering scalable solutions that complement traditional therapies [9, 10]. Meta-analyses confirm their effectiveness in reducing depression, particularly in WEIRD countries, by providing flexible, location-independent support [11, 12]. Integrating these interventions could significantly expand mental health care’s reach, ensuring more equitable access to effective treatments globally [13]. However, previous studies suggest that unguided IBIs, which means having no human support at any stage, tend to be associated with higher dropout rates [14], lower uptake rates (i.e., logging into an intervention) [15, 16], lower adherence (e.g., completing modules of an intervention) [17], and sometimes also lower effects [18].

In some of the studies mentioned above, the authors propose that other forms of human interaction (such as peer-to-peer support online forums) might be beneficial for the treatment of depression [19]. In one of the meta-analyses conducted on peer support interventions for depression, evidence was found that peer support in the form of internet support groups (ISGs)/discussion groups and chat rooms led to a reduction in depressive symptoms relative to usual care [20]. It could be assumed that improvements in coping strategies, both in social interactions and regarding their health condition [21], might lead to increased supportive communication [22], which in turn could significantly enhance adherence to treatment and reduce drop-out rates [23]. However, one of the studies conducted by Andersson et al. [24] showed that there were no improvements seen in depressive symptoms due to discussion groups in the people suffering with depression. Therefore, we need more research in this direction of examining the association between IBIs using peer-to-peer support online forums as compared to the IBIs without using peer-to-peer support online forums in the treatment of depression using a randomized controlled trial to understand it better.

Furthermore, many of the studies that used IBIs for depression have been conducted on the WEIRD countries and only a few studies have been conducted in the non-WEIRD countries [25–27]. Hendriks et al.’s [28] review highlighted that most randomized controlled trials (RCTs) examining the effects of IBIs on depression originated from Western countries, with only 22% from non-Western countries, and these findings based on WEIRD populations may not generalize entirely to all cultures or ethnicities. To our knowledge, none of the studies have investigated the intercultural difference based on the usage of peer support online forums between the WEIRD and non-WEIRD population. Therefore, one of the main aims of our study is to investigate whether IBIs with peer-to-peer support online forums could improve the symptoms of depression as compared to the IBIs without peer-to-peer support and, in addition, to examine the cultural differences in the use of peer-to-peer support online forums between India and Switzerland as these countries have different cultures.

In the previous study conducted in India, traditional support structures, such as extended families and community groups, might influence how peer support is perceived and utilized [29]. Integrating these traditional structures into an online intervention could enhance its effectiveness. In contrast, in Switzerland, there is a strong emphasis on independence but also strong social support among friends [30, 31]; therefore, assessing peer support in the Swiss context and in comparison to India will extend our knowledge by understanding and addressing these cultural differences, IBIs with online peer support interventions for depression that could be tailored to meet the unique needs and preferences of participants in both India and Switzerland, potentially enhancing their effectiveness and acceptance. These cross-cultural comparisons will also contribute to guide the policymakers in creating inclusive and effective mental health policies that can be adapted across different cultures.

In this study, we will use the IBI for depression called Deprexis with peer-to-peer support and without peer-to-peer support in the French and English language through a randomized clinical trial. Deprexis is an integrative intervention that is broadly based on cognitive-behavioral therapy and has been tested in German-speaking countries [32–36], the United States [37], Brazil [38], and the German-speaking part of Switzerland [33]. This study will be the first to test its efficacy in French in Switzerland.

In addition, previous studies have evidenced that a robust reward system is linked to feelings of pleasure, emotional regulation, and overall mood stability, enhancing the ability to cope with stress and acting as a buffer against mental health issues [39–41]. Anhedonia, a core symptom of depression, involves a loss of pleasure and is often associated with dysfunction in the reward system [42, 43]. Studies using monetary rewards indicate that individuals with depression show reduced positive responses and decreased reward-seeking behavior [44], as evidenced by their performance on tasks like the Iowa Gambling Task [42]. In addition, individuals with depression frequently display deficits in social functioning and diminished motivation to engage in social interaction [45]. In one of our previous studies using the Fribourg Reward Task [46–48], reduced mood responses to monetary rewards were observed in individuals with pain symptoms in Switzerland but not in India [47, 48]. In addition, social reward might also represent a particularly salient category of reward stimuli contributing to depression [49]. Limited empirical research has been conducted to show the relationship between depression and social rewards [50]. Our study aims to further explore reward responses in individuals with depressive symptoms using the Fribourg Reward Task that includes three reward conditions (monetary, social, and no-reward) in India and Switzerland, examining intercultural differences in social and monetary rewards and testing the effects of an online treatment on these reward responses.

At the end, we also plan to conduct exploratory analyses to investigate the effects of treatment on comorbid symptoms of depression, such as stress and anxiety, which are highly comorbid with depression [50, 51]. Additionally, we will assess the impact of positive outcomes like self-efficacy, quality of life, and self-esteem on depression symptoms, as improvements in these areas have been shown to reduce depression [52–54]. We will also investigate the influence of trauma and pain on depression symptoms [55, 56]. Lastly, we aim to evaluate client satisfaction, usability, and working alliance based on technology-related measures.

### The goal of the present study

The present study, registered at ClinicalTrials.gov (NCT06480474) and Swiss National Clinical Trials (SNCTP000005917), aims to investigate (1) whether Deprexis with peer-to-peer support online forums could improve the symptoms of depression as compared to Deprexis without peer-to-peer support, (2) to examine the cultural differences in the use of peer-to-peer support online forums between a non-WEIRD country (India) and a WEIRD country (Switzerland), and (3) to examine the efficacy of Deprexis in the French-speaking part of Switzerland, and to assess the effects of the online program on mood responses to monetary and social rewards in individuals with depressive symptoms in India and Switzerland.

This study has two experimental conditions (Deprexis and Deprexis with an online forum) compared with a control condition (waiting list). The control group is used to assess the difference between having access to an intervention and having no access. We expect a superiority of Deprexis and Deprexis with the online forum group against the waiting list group (control group) and a superiority of Deprexis with the online forum group against the unguided Deprexis group. Additionally, we expect higher adherence to Deprexis with the online forum. The control group will be placed on a waiting list and will have access to the program after the initial 8 weeks.

## Methods

### Study design

This study is conducted as a randomized controlled trial. Participants will be randomly assigned to either of the three groups. The first group will use Deprexis, an internet-based self-help treatment based on cognitive-behavioral therapy, for 8 weeks. The second group will have access to Deprexis and a peer-to-peer support online forum for sharing and support. The third control group will access Deprexis after the initial 8 weeks. In total, 240 participants will be recruited, with 80 participants in each group.

### Participants

Participants will be recruited through flyers and emails, word-of-mouth, social media (e.g., Facebook, Instagram), depression-related websites, private psychotherapists, outpatient clinics in French-speaking Switzerland, and the English-speaking population from India. Interested participants will receive information on the study, and then they will be given at least 24 h to confirm their participation by signing and returning the informed consent of the study (via email). They will be screened to assess eligibility using a rapid eligibility check, using the inclusion and exclusion checklist and the Patient Health Questionnaire (PHQ-9; [57]).

The inclusion criteria are (1) providing signed informed consent; (2) being at least 18 years of age; (3) having access to a computer/laptop/tablet/smartphone with an internet connection; (4) having sufficient French language skills in Switzerland and English in India; (5) meeting criteria for mild to moderate depression as operationalized by the Patient Health Questionnaire-9 (PHQ-9 = 5–14) (PHQ-9; [57]) meeting the diagnostic criteria of the characterized depressive disorder according to the diagnostic interview by telephone; and (7) providing the emergency contact before treatment.

However, they will be excluded from the study if they present any of the following criteria: (1) having active suicidal plans (if they have a score of 2 or higher on the suicide item of the PHQ-9 [57] or with active suicidal plans in the diagnostic telephone interview; (2) having a history of psychotic disorder or bipolar disorder; and (3) changing their dosage of prescribed medication for anxiety or depression in the last month before the study. When potential participants fulfill inclusion criteria, they will be interviewed by telephone for screening using the MINI International Neuropsychiatric Interview (M.I.N.I; [58]). The current protocol is prepared in accordance with the “Standard Protocol Items: Recommendations for Interventional Trials (SPIRIT) 2013” guidelines [59].

### Randomization

After checking the inclusion criteria, a weighted randomization procedure is used in which 240 participants (80 participants each) are assigned in a 1:2:2 probability to one of the three groups (Deprexis group, Deprexis and a peer-to-peer support online forum, and control group). The random allocation will be done by a randomization module in the REDCap software and will be unknown to the investigators. This clinical trial includes measurement at baseline (T0), at mid-intervention (i.e., 4 weeks, T1), at post-intervention (i.e., 8 weeks, T2), and at follow-up (T3), 3 months after the intervention.

### Description of the intervention

#### Deprexis

Deprexis is the internet-based self-help program that we will use in this study and is based on different psychotherapeutic approaches, consistent with a cognitive-behavioral perspective [32]. Deprexis has been shown to be efficacious in several previous clinical trial studies (e.g., [35, 36, 60]). Deprexis contains 10 modules that all participants can access at any time. Users of the program are encouraged to repeat all modules as many times and as often as they wish, after they have completed the full module sequence. A brief description of each module is described below:

1. *Introduction:* Introducing the program’s function and purpose; psycho-educational overview of cognitive-behavioral therapy (CBT) model of depression (e.g., relationships among thoughts, behaviors, and feelings); exploring perceived reasons for depression; brief mindfulness exercise: learning to calmly observe the flow of thoughts and feelings; worksheet to encourage regular program use; prompting to complete mood questionnaires.
2. *Behavioral activation:* This module includes standard behavioral activation and procedures used in different manuals, with some modifications. Users are encouraged to set activities that may satisfy basic psychological needs (e.g., social relatedness, competence, self-esteem, hedonic enjoyment).
3. *Cognitive modification:* Similar to the first module, this one also includes standard cognitive interventions with some adaptations for the online format of the program. There is a specific focus on the mood-determining role of automatic thoughts, interactions between thoughts, emotions, etc., or techniques on acceptance.
4. *Mindfulness and acceptance:* This module includes brief exercises to help suppress unwanted thoughts and feelings, or to learn to deal with unwelcome experiences with calmer and acceptance.
5. *Interpersonal skills:* This module focuses on problems linked to interpersonal adjustment, it offers several suggestions to help the users to improve their interpersonal adjustment, such as tips for improved verbal and nonverbal communication, or guidelines for relationship-enhancing behaviors.
6. *Relaxation, physical exercise and lifestyle modification:* This module includes relaxation exercises, repeated tension exercises, healthy lifestyle tips, using imagery and breathing exercises.
7. *Problem solving: *This module includes exercises to teach a problem-solving approach linked to depression-related problems (including how to define problems in concrete terms, set achievable goals or generate solutions, etc.).
8. *Childhood experiences and early schemas:* In this module, techniques to deal with difficult childhood memories are provided. For instance, users learn expressive writing, forgiveness, or acceptance of difficult memories.
9. *Positive psychology interventions:* This module focuses on positive psychology based on positive experiences (e.g., happiness, well-being, life satisfaction). Exercises such as learning to enjoy positive experiences and memories, to satisfy basic needs or to cultivate strengths and talents are provided.
10. *Dreamwork and emotion-focused interventions:* This module contains information and techniques on dream-related work and emotions. It offers techniques and exercises such as writing a dream journal, working on problem-laden dreams with positive endings or making links between dream contents and real-life problems. This module is offered for users who show positive attitudes toward such content.

The Deprexis program can be accessed from regular internet browsers, via a laptop, computer, tablet, or smartphone, through a secure website. Each participant will be provided with a password-protected account. Participants’ use of Deprexis will be automatically recorded by the program, which will allow for an automated measure of treatment compliance. Also, this program has been standardized and validated for 90 days based on evidence-based studies [32, 61]. Participants will have full access to the medical device and must first go once through all modules and are then free to repeat any module, as often as they wish. Participants are informed that they should work 30 min at least 1–2 times a week on the program. Participants can receive optional automatic reminders within the program, which also provide brief motivational-supportive messages. Moreover, participants in the experimental condition of medical devices will receive regular messages on the forum (described below) from the moderator, to encourage the use of the program and the online forum.

#### Peer-to-peer support online forum

In addition, participants in one of the experimental groups will have access to an online forum, which will be built for the study. This forum will consist of a general discussion page, in which participants will be able to ask and answer questions, and for this, the participants will be divided into a group of 10 each to share their experiences in a group if they wish to. Additionally, there will be different subforums that will each be related to specific modules of Deprexis. Participants will share their experiences and if they have any questions related to the module. A moderator will regularly check that no personal information or sensitive information is disclosed, and will once a week ask some questions on the forum to encourage participants to contribute to the forum. The moderator is not a psychotherapist and will not answer questions related to the program or advise participants on the intervention.

### Procedure

This study will be entirely online. All participants will undergo the same screening procedure, including a standardized questionnaire (PHQ-9; [57]) and a structured clinical psychiatric interview (MINI; [58]) performed via telephone. Assessments will be performed by trained research assistants from the University and supervised by a clinician from our department. Eligible participants will be randomized into one of three groups of 80 participants each: one with Deprexis only, one with Deprexis and peer-to-peer support online forum, and a control group on a waiting list, which will access Deprexis after 8 weeks.

**Fig. 1:**
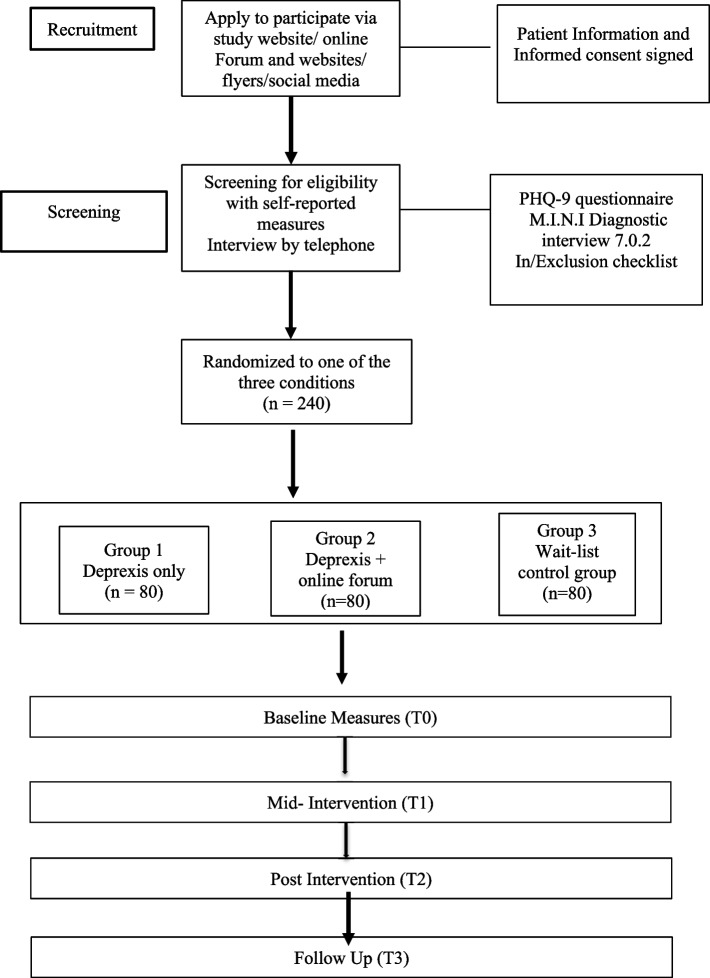
Flow chart of the study

The first group will use Deprexis, an internet-based self-help treatment based on cognitive-behavioral therapy, for 8 weeks. The second group will have access to Deprexis and a peer-to-peer support forum for sharing and support. The third control group will access Deprexis after the initial 8 weeks. Measurements will be taken at baseline (T0), mid-intervention (4 weeks, T1), post-intervention (8 weeks, T2), and follow-up (3 months after intervention, T3) (see Table 1). Participants will complete online questionnaires on RedCap (https://redcap.unifr.ch) and an adapted online version of the Fribourg reward task [46–48] at different study points (see Fig. 1).

**Table 1:** This table demonstrates different psychometric measures used in the study

Study Periods	Screening	Baseline measures (T0)	Mid-intervention (T1)	Post-intervention (T2)	Follow-up (T3)	“End-of-study” visit
Visit	1	2	3	4	5	6
Time (weeks/day)	−2 weeks- 0	0	+ 4 weeks ± 2 weeks	+ 8 weeks ± 2 weeks	+ 20 weeks ± 2 weeks	Any time after completion or withdrawal
Patient information sheet	x					
Informed consent (after at least 24 h)	x					
PHQ-9 (including suicidal risks)	x	x	x	x	x	
MINI	x					
In-/exclusion criteria checklist	x					
SHAPS		x	x	x	x	
BIS/BAS		x	x	x	x	
MSPSS		x	x	x	x	
GAD-7		x	x	x	x	
SF-12		x	x	x	x	
RSES		x	x	x	x	
GSES		x	x	x	x	
PSS		x	x	x	x	
SSS-8		x	x	x	x	
PCL-5		x	x	x	x	
CTQ-SF		x	x	x	x	
SUS				x		
CSQ			x	x		
NEP			x	x	x	
Fribourg Reward Task		x	x	x		

*PHQ-9* Patient Health Questionnaire, *MINI* MINI International Neuropsychiatric Interview, *SHAPS* Snaith-Hamilton Pleasure Scale, *BIS/BAS* Behavioral Inhibition/Behavioral Activation System, *MSPSS* Perceived Social Support, *GAD-7* Generalized Anxiety Disorder Scale, *SF-12* Short-Form Health Survey, *RSES* Rosenberg Self-Esteem Scale, *GSES* General Self-Efficacy Scale, *PSS* Perceived Stress Scale, *SSS-8* Somatic Symptom Scale, *PCL-5* Post-Traumatic Stress Disorder Checklist for DSM-5, *CTQ-SF* Childhood Trauma Questionnaire-Short Form, *SUS* System Usability Scale, *CSQ* Client Satisfaction Questionnaire, *NEP* Negative Effects of Psychotherapy

All data will be encrypted and identifiable only by a code not linked to the participant’s identity. The only real-time interaction with staff will be during scheduled telephone diagnostic interviews at pre-, post-, and follow-up assessments. No spontaneous calls will be made unless safety concerns arise, such as severe suicidal intentions reported by a participant. If the participant is assessed as high risk at the time of the screening, the person concerned will not be admitted to the study and we will (a) explain to them why they are excluded from the study, (b) ask them to provide us the contact information of a close person, and (c) also provide them the list of therapists as well as phone numbers of all psychiatric emergencies in all French-speaking cantons and of our internal centers as well as of Centre Psychosocial of the Réseau fribourgeois de santé mentale (RFSM) in Fribourg. If they withdraw from the study, the data collected up to this point will be used for further analysis since it is important for the study’s scientific value. Also, for each included participant, we will elaborate with them an individualized safety plan after they have been included for the case their symptoms worsen during the course of the study. The safety plan will include the fact that 1) they should inform us if this happens, which persons or professional help should be contacted, and that they also can get support from the study team on the phone.

Adherence strategy: To encourage study adherence, participants will receive compensation at various stages of the study. While there is no direct compensation for engaging with the Deprexis program, participants will receive the monetary rewards earned during the Fribourg reward task at T0 and T2 (maximum CHF 24) and an additional CHF 10 for completing the questionnaires at each time point (T0, T1, T2), for a total of CHF 30. To further support participation, psychology students at the University in Switzerland can opt to receive experimental credits instead of monetary compensation (2 credits per time point, up to a maximum of 6 credits). By offering structured financial incentives and academic credits, the study aims to enhance adherence by maintaining participants’ engagement and ensuring completion of all study phases.

### Study outcome measures

All outcome measures will be assessed online with validated French versions (for Swiss participants) and English versions (for Indian participants) of the original questionnaires.

#### Primary outcome measures

Patient Health Questionnaire (PHQ-9; English version [62]; French version [57]). This self-reported measure is used in several RCT studies to assess depressive symptoms [34, 36]. The PHQ-9 is a valid, reliable, and useful tool for clinical and research purposes.

#### Secondary outcome measures

##### Fribourg reward task

In parallel, participants will be asked to complete an online reward task, to measure reaction times, accuracy, and mood reactions to both monetary and social rewards. For this, we will use an online adapted behavioral version of the Fribourg reward task [46–48]. This task was originally programmed using E-Prime software (version 1.1.3, Psychology Software Tools Inc., Pittsburg, Pa., USA) and made available online using OpenSesame, a graphical experiment builder for the social sciences. The experimental task was presented in three block conditions, comprising reward conditions (monetary reward, social reward, and no reward). For this task, there are three conditions (monetary reward, social reward, and no reward). Each condition consists of 12 trials, and the order of the blocks was pseudo-randomized. In the three-reward conditions, at the onset of each trial, a visual cue (2000 ms) was presented (3 yellow circles), along with the reward associated with performance. After the presentation of a fixation cross (500 ms), participants saw an array of yellow circles (3 circles, 2000 ms). A fixation cross (3000 ms) was presented before the visual target. The visual target (a green circle, 3000 ms) was displayed in any position on the screen and signaled that the participant should decide as quickly as possible whether this circle was in the same position as one of the circles presented previously. After response execution and a variable jittered interstimulus interval (ISI; 0 ms or 2000 ms), the feedback screen (1500 ms) informed the participant of their winnings. In each condition, the way correct and incorrect trials are presented differs as follows:

- In the social reward condition: a neutral face is presented for incorrect trials and a smiling face for correct trials.
- In the monetary reward condition: a screen with “CHF 0” is shown for incorrect trials and “CHF 1” for correct ones.
- In the control condition: a blank screen is presented in both correct and incorrect trials.

At the end of all trials, a feedback screen (1000 ms) indicating the cumulative amount of monetary reward or social reward (smileys) earned (in the monetary and social reward conditions) or a blank screen in the no-reward condition was presented. Correct responses were associated with monetary gains (“CHF 10”) in the monetary reward condition. Correct responses were not associated with any gains in the no-reward condition. We asked participants to rate their momentary mood and stress level using a visual analog scale from 0 (bad mood)—10 (good mood), with smileys at the anchor points (0 =; (10 =). Participants rated their momentary mood and stress level on a scale of 0 to 10 at baseline, at the beginning of the experimental session, and before and after each block for a maximal duration of the 20 s. Participants were informed that they would receive the total sum in cash at the end of the session. Participants underwent a training phase before proceeding to the main task. A criterion of 70% correct responses was chosen to prevent arbitrary guessing and thereby verify understanding of the task and ensure that participants would win similar amounts of money.

Snaith-Hamilton Pleasure Scale (SHAPS; English version [63, 64]; French version [63]). The SHAPS measures the ability to experience pleasure. It covers four different areas of hedonic experience: interest/pastimes, social interaction, sensory experience, and food/drink. The scale has good internal consistency in both English and French and correlates significantly with other measures of effect and personality [62, 63].

Behavioral Inhibition System/Behavioral Activation System (BIS/BAS; English version [65]; French version [66]). This scale is a self-reported questionnaire, which measures two aspects linked to personality, that is the activation and the inhibition systems, or sensitivity to punishment and reward. The BIS/BAS scales show good internal consistency in both subscales [67].

Perceived Social Support (MSPSS; English version [68]; French version [69]). The PSS is a self-report questionnaire, which assesses the perception of support from family, friends, and a significant other. The PSS scale shows good psychometric properties with good internal consistency in both English and French [67, 68].

#### Moderators and mediators

Generalized Anxiety Disorder Scale (GAD-7; English version [70]; French version [71]). The GAD-7 measures general anxiety symptoms, such as feeling nervous, worried, having trouble relaxing, restlessness, feeling annoyed or irritable, and feeling afraid that something awful might happen. Internal consistency of the GAD-7 is good in both the original and French versions, and it has good convergent validity with other anxiety scales [72].

Short-Form Health Survey-12 (SF-12; English version [73]; French version [74]). Quality of life is assessed with the SF-12. Two subscales measure respectively physical and mental aspects of health-related quality of life. The SF-12 shows good psychometric properties, roughly equivalent to the long form, the SF-36, and is widely used as an estimate of the general quality of life [72].

Rosenberg Self-Esteem Scale (RSES; English version [75]; French version [76]). The RSE scale is a self-report scale that measures one’s overall sense of worthiness as a person and has been translated and used worldwide, indicating that self-esteem is a global measure across cultures [77].

General Self-Efficacy Scale (GSES; English version [78]; English version [79]). This trait scale assesses the belief in one’s competence to cope with challenging demands. Its internal consistency is good and has been shown to be a universal construct across 25 countries [77, 78].

Perceived Stress Scale-Short Version (PSS; English version [80]; French version [81]). The PSS scale is a widely used psychological instrument to measure perceptions of stress. It is a self-reported questionnaire. The internal consistency of the scale is good in both French and English [79].

Somatic Symptom Scale (English version [82]; French version [83]). This scale is a brief self-report questionnaire used to assess somatic symptom burden. It measures the perceived burden of common somatic symptoms. The internal consistency of the scale is good in both French and English [82].

Post-Traumatic Stress Disorder Checklist for DSM-5 (PCL-5, English version [84]; French version [85]). The PCL-5 is a 20-item questionnaire, corresponding to the DSM-5 symptom criteria for PTSD. The scale ranges from 0 to 4 for each symptom from “Not at all,” “A little bit,” “Moderately,” “Quite a bit,” and “Extremely.” The PCL-5 shows good psychometric properties and is widely used as an estimate of the general quality of life [83].

Childhood Trauma Questionnaire-Short Form (CTQ-SF; English version [86]; French version [87]). The CTQ-SF is a retrospective, self-report measure that was developed to provide a brief, reliable, and valid assessment of a broad range of traumatic experiences in childhood. It assesses experiences of abuse and neglect in childhood, including physical, emotional, and sexual abuse and physical and emotional neglect, as well as related aspects of the child-rearing environment. The CTQ-SF is intended for adolescents and adults. It contains 5 items arranged according to four factors: physical and emotional abuse, emotional neglect, sexual abuse, and physical neglect. Responses are quantified on a 5-point Likert-type scale according to the frequency with which experiences occurred, with 1 = “never true” and 5 = “very often true.” The CTQ has good psychometric properties which make it a reliable and valid measure for assessing CTQ’s in English and French-speaking populations, both for research purposes and clinical practice [88].

#### Treatment characteristics

System usability scale (SUS; English version [89]; French version [90]). The SUS allows the evaluation of the user experience and the satisfaction related to the use of a product. This scale has been widely used since the beginning [91]. The scale presents a good internal consistency [88, 89].

Client Satisfaction Questionnaire (CSQ; English version [92]; French version [93]). The CSQ-8 is a self-report questionnaire that assesses the general level of satisfaction with the service received. It was originally developed to measure satisfaction with inpatient treatment. In this study, we will use a version that was slightly adapted for internet-based treatments, and that has shown good internal consistency in previous studies [33, 94].

Negative Effects of Psychotherapy (NEP; [95]). This is a tool used to assess and measure various negative occurrences or consequences that can arise from psychological interventions or treatments. The primary purpose of the scale is to systematically evaluate and quantify adverse outcomes that may result from receiving psychological treatment. The scale covers a broad spectrum of potential negative incidents, such as intensification of symptoms, unintended side effects of treatment, client dissatisfaction or disillusionment, therapist-client relationship strains, miscommunication or misunderstandings, ethical concerns or breaches. Items on the scale are typically developed through empirical research and clinical observations to ensure they adequately capture the diverse array of negative incidents that could occur during or as a result of psychological treatment [94].

### Power analysis

We plan to include 240 participants based on a priori power analysis. With an α error level of 0.05, statistical power of 0.80, and a correlation of *r* = 0.5 between baseline and 8-week intervals, we need 174 participants (G*Power). Considering a dropout rate of 13% (based on [96]), we account for 23 participants dropping out. Additionally, given an odds ratio (OR) of 2.88 for 2-week prevalence [66], and anticipating 20% exclusion post-signing the consent form, we estimate needing 240 participants. They will be divided into three groups of 80 each (see Fig. 1).

### Planned statistical analyses

The analysis will be done with the help of appropriate software programs (e.g., JASP or SPSS) by the end of the study. It will be conducted on the Intention-To-Treat (ITT) sample. Before analysis, all outcome variables will be tested for normality using the Kolmogorov-Smirnov test and, if necessary, transformed to obtain a normal distribution. To test for the main and interaction effects of treatment components on primary and secondary outcomes, a linear mixed-model repeated measures ANOVA with time as a within-groups factor and treatment condition as a between-groups factor will be used for the main research question. Also, repeated measures ANOVA will be used to test the effects of Deprexis on reward responses and on perceived social support in relationship to the forum utilization. We will also use mediation analyses to test our objectives. If the questionnaires have more than 30% of the missing data, they will not be used in the analysis, and for the other cases, missing data will be computed with the help of the statistics software. Interim analysis will be performed for preliminary data presentation at scientific meetings and to check data quality.

### Trial status

The study was registered at ClinicalTrials.gov (NCT06480474) and Swiss National Clinical Trials (SNCTP000005917). The Ethics committee at the Canton of Vaud (CER-VD) approved the study in May 2024 (Protocol Date: 22.05.2024; Version: 4; 2023-D0112). Recruitment began in June 2024 and is expected to conclude in May 2026. As of August 2025, 28 participants have been recruited, of whom 9 have completed the post-intervention (T2) assessment and 6 have completed the follow-up (T3) assessment.

### Data Safety Monitoring Committee

As to the best of our knowledge and based on numerous similar clinical trials using Deprexis for depression (e.g., [35, 36, 60]), it does not bear any significant risk to the participants. All data are encrypted, and the Sponsor-Investigator and the associated investigators warrant data and participant safety. We will use Redcap for data collection and management. Monitoring will be performed internally at the Department of Psychology by a professor, who is also an expert in online psychotherapy and conducting clinical trials in that field, and at the same time having the necessary Good Clinical Practice (GCP) qualifications.

## Discussion

Our study delves into evaluating the effectiveness of Deprexis with peer-to-peer support online forums vs. without peer-to-peer support online forums in alleviating depressive symptoms, in addition emphasizing the cultural differences between WEIRD (Western, Educated, Industrialized, Rich, and Democratic) and non-WEIRD countries, specifically Switzerland and India. Our study aligns with existing literature on the benefits of peer support in managing depression. Some of the studies conducted using peer-to-peer support online forums have shown to improve coping strategies, social interaction, and adherence to treatment, while reducing dropout rates and depressive symptoms [19–23]; however, more studies are needed in this direction, and in addition, there is a gap in literature related to its cross-cultural differences. Our study underscores significant cultural differences in the use and efficacy of peer support online forums between India and Switzerland. In India, mental health support is deeply rooted in traditional support structures like extended families and community groups [29]. Incorporating these elements into online interventions could amplify their effectiveness. Conversely, Switzerland values independence alongside strong social support from friends [30, 31], suggesting a unique approach to peer support that could benefit mental health interventions. This study is groundbreaking in examining the effectiveness of peer-to-peer support online forums across these two cultural contexts.

Additionally, this research marks the first exploration of Deprexis’s efficacy in French-speaking Switzerland and in India. Previous studies have validated Deprexis’s effectiveness in German-speaking regions [32–36], the USA [37], and Brazil [38]. Our findings aim to extend its proven benefits to the French-speaking demographic, broadening its application.

The study also investigates mood responses to monetary and social rewards using the Fribourg Reward Task, shedding light on the relationship between depression and reward system dysfunction. Consistent with prior research, individuals with depressive symptoms exhibit reduced responses to monetary and social rewards [42, 43]. By exploring these dynamics in a cross-cultural context, our study emphasizes the necessity of cultural considerations in mental health interventions.

The implications of this study are profound for mental health policy and practice. Demonstrating the effectiveness of peer support online forums and the Deprexis program across diverse cultural settings supports the need for culturally adapted mental health interventions. Policymakers should consider integrating these scalable, evidence-based solutions into existing mental health systems, particularly in non-WEIRD countries, to enhance access and close treatment gaps.

Moreover, this research provides valuable insights into comorbid symptoms of depression, such as stress and anxiety, and positive outcomes like self-efficacy, quality of life, and self-esteem. Understanding these factors can lead to more effective, personalized interventions. Given the high prevalence of depression and the barriers to traditional mental health services, this study has the potential to significantly impact public health by offering accessible, scalable solutions for managing depression.

### Limitation

While the study provides valuable insights, several limitations should be acknowledged. They are as follows: (1) The reliance on self-reported data may introduce bias, (2) The online intervention faces several challenges, including limited personal interaction, which may affect user engagement. The absence of direct human interaction can be significant, yet we mitigate this by providing access to a peer-to-peer support online forum where participants are encouraged to share their experiences. (3) Engagement and adherence could also be problematic as participants might lose motivation or face distractions. To address this, a moderator in the online forum will regularly encourage participation and send reminders to complete the Deprexis modules. 4) The program requires a high degree of self-motivation and discipline, potentially difficult for some users; however, peer-to-peer support in the online forum aims to enhance motivation. 5) Technical barriers are another concern, requiring reliable internet access and basic technical skills. We ensure that participants have access to necessary technology, though those less familiar with digital platforms may still struggle. 6) Privacy and confidentiality are paramount, as users may hesitate to engage fully if concerned about data security. Our study addresses this by protecting access with passwords, encrypting data transmission, and ensuring no identifiable information is stored. Moderators will also delete any personal information inadvertently shared by participants.

## Conclusion

This study highlights the potential of IBIs, including peer-to-peer support online forums and the Deprexis program, to address the global burden of depression. By examining cultural differences in the use and effectiveness of these interventions, the study provides a foundation for developing more inclusive and effective mental health strategies. The findings from this study underscore the importance of culturally adapted interventions and support the integration of online mental health care into existing health systems to reduce treatment gaps and improve mental health outcomes worldwide. Future research is needed to explore the effectiveness of these interventions in other cultural settings and examine long-term outcomes.

## Supplementary Information

Supplementary Material 1.

## Data Availability

The datasets that will be used and/or analyzed during the current study will be made available from the corresponding author on reasonable request.
